# Integrative AI driven microbiome analysis for optimizing sports nutrition and enhancing athletic performance through personalized dietary interventions

**DOI:** 10.3389/fnut.2026.1754203

**Published:** 2026-06-12

**Authors:** Yankun Zhang, Hongjing Wang, Ting Deng

**Affiliations:** 1 School of Physical Education, Suihua University, Suihua, China; 2 School of Special Education, Suihua University, Suihua, China; 3 School of Electrical Engineering, Nanjing Normal University, Nanjing, China

**Keywords:** athletic performance, dietary recommendations, IMAPON, microbiome composition, personalized nutrition

## Abstract

**Introduction:**

The relationship between microbiome composition, athletic performance, and personalized nutrition offers significant potential for optimizing sports nutrition and enhancing athletic outcomes through tailored dietary strategies.

**Methods:**

This study presents a novel framework, Integrative Microbiome Athletic Performance Optimization Network (IMAPON), designed to address this challenge by integrating microbiome data, athletic performance metrics, and demographic and physiological information to generate precise dietary recommendations. IMAPON consists of three core modules: the Microbiome Feature Extraction Module (MFEM), the Athletic Performance Prediction Module (APPM), and the Personalized Dietary Recommendation Module (PDRM). Two innovative strategies, the Adaptive Feature Integration Strategy (AFIS) and the Performance Driven Optimization Strategy (PDOS), are incorporated to improve system efficacy. AFIS facilitates dynamic integration of features from heterogeneous data sources, while PDOS aligns dietary interventions with specific athletic performance objectives. The framework employs advanced computational techniques, including feature extraction, representation learning, and optimization, formalized through mathematical models to capture latent interactions between microbiome composition, physiological factors, and performance metrics.

**Results and discussion:**

Experimental results demonstrate the effectiveness of IMAPON in generating actionable dietary recommendations, highlighting its potential to transform sports nutrition by enabling precise, data driven interventions tailored to individual athletes. This approach represents a significant advancement in leveraging artificial intelligence for personalized nutrition and athletic performance enhancement.

## 1 Introduction

The study of microbiome analysis in optimizing sports nutrition and enhancing athletic performance through personalized dietary interventions has garnered significant attention due to its potential to revolutionize athletic training and health management. Not only does the human microbiome play a critical role in digestion and nutrient absorption, but it also influences immune function, inflammation, and energy metabolism, all of which are vital for athletes (1). Furthermore, personalized dietary interventions tailored to an individual's microbiome composition can address the limitations of generalized nutrition plans, offering more precise strategies to improve performance and recovery (2). This task is not merely about understanding the microbiome but also about leveraging advanced AI driven methodologies to analyze complex microbiome data and translate findings into actionable dietary recommendations (3). By integrating AI into microbiome research, researchers can uncover hidden patterns, predict outcomes, and optimize interventions, thereby advancing both scientific understanding and practical applications in sports nutrition (4).

In the initial stages of microbiome analysis, researchers primarily focused on manually encoding domain specific knowledge into structured systems. These early methods aimed to model the interactions between microbiome components and their effects on human health by using predefined rules and logical frameworks (5). While these approaches provided valuable insights into causal relationships, they were often limited by their scalability and adaptability, as they required significant manual input and struggled with the complexity of microbiome data (6). Moreover, these systems were not well suited for generalizing across diverse datasets, which posed challenges for developing personalized dietary interventions (7). As the field evolved, researchers began to explore more flexible approaches that could handle larger and more complex datasets. The introduction of algorithms capable of learning from structured feature sets marked a significant shift in microbiome analysis (8). Techniques such as support vector machines and random forests allowed for the analysis of high dimensional data, uncovering patterns that were previously undetectable (9). These methods facilitated the integration of various data sources, including genetic and environmental factors, to create comprehensive models of microbiome function (10). However, despite these advancements, the reliance on extensive feature engineering and labeled data remained a significant limitation, as it was both costly and time consuming (11).

To further advance the field, the adoption of deep learning models has provided new opportunities for analyzing complex and unstructured microbiome data. Architectures like convolutional neural networks and recurrent neural networks have shown exceptional capabilities in extracting features and uncovering insights into microbial interactions (12). The use of pre trained models and transfer learning has further enhanced the ability to adapt existing models to specific microbiome tasks, reducing the dependency on large labeled datasets (13). Despite these advancements, deep learning methods often require substantial computational resources and face challenges related to interpretability, which can hinder the translation of findings into practical dietary recommendations (14). These ongoing challenges underscore the need for innovative solutions that integrate the strengths of previous methodologies while addressing their limitations (15).

Based on the limitations of existing symbolic AI, machine learning, and deep learning methods, an integrative AI driven framework for microbiome analysis is proposed to optimize sports nutrition and enhance athletic performance through personalized dietary interventions. The framework is primarily grounded in data driven learning, while drawing inspiration from symbolic AI to improve interpretability and facilitate the incorporation of domain knowledge. The predictive capability of machine learning and the representation learning strength of deep learning are leveraged to model complex interactions among microbiome composition, physiological factors, and athletic performance metrics. By integrating domain knowledge with advanced data driven techniques, the proposed approach addresses scalability and generalization challenges in previous methods while providing actionable insights for personalized nutrition. Furthermore, multi modal data sources, including genetic, environmental, and lifestyle factors, are incorporated to construct a more comprehensive representation of microbiome related processes. This integrative design enhances the accuracy and robustness of dietary recommendations and improves their applicability across diverse athletic populations and scenarios. Although the current implementation focuses on data driven modeling, the framework is designed to be extensible to future incorporation of explicit knowledge guided or symbolic components for improved interpretability. This approach represents a step toward unified, multi modal, and knowledge aware AI systems for sports nutrition and microbiome research.

The main contributions of this work are summarized as follows:

- An integrative AI driven framework is proposed for microbiome informed sports nutrition analysis, which leverages data driven learning while being compatible with domain knowledge integration to address limitations of existing approaches.
- A multimodal modeling strategy is developed to capture interactions among dietary intake, physiological factors, and microbiome related features, enabling more structured and personalized nutritional recommendations.
- Experimental results on dietary intake estimation benchmarks demonstrate the effectiveness of the proposed representation and regression framework, providing a practical component for downstream personalized nutrition modeling.

## 2 Related work

### 2.1 AI driven microbiome data analysis

The application of artificial intelligence in microbiome research has significantly advanced the ability to analyze complex datasets, providing insights into the intricate relationships between microbial communities and host physiology. Machine learning algorithms, including deep learning models, have been utilized to process metagenomic and metabolomic data, revealing microbial structures and functional pathways (9). Convolutional neural networks have demonstrated their utility in classifying microbial taxa with high precision, while recurrent neural networks have been employed to predict functional profiles (10). Unsupervised learning techniques, such as clustering methods, have contributed to identifying novel microbial signatures linked to physiological outcomes (12). The integration of microbiome data with other omics layers, such as proteomics and genomics, has enabled researchers to explore host microbiome interactions comprehensively (13). Multi omics approaches have been particularly impactful in sports nutrition, where microbial pathways influencing nutrient metabolism and energy production are of interest (14). Studies have highlighted the role of gut microbes in enhancing the bioavailability of essential amino acids, which are critical for muscle repair and growth (15). Predictive models developed through AI have facilitated personalized dietary interventions, optimizing nutrient absorption and recovery times (16). The scalability of AI driven microbiome analysis continues to expand its applications in personalized nutrition and athletic performance (17).

### 2.2 Personalized nutrition for athletes

The concept of personalized nutrition has gained prominence in sports science, focusing on tailoring dietary strategies to individual variability in microbiome composition and metabolic responses. The gut microbiome's influence on nutrient digestion and absorption underscores its importance in this domain (18). Research has identified microbial biomarkers that correlate with dietary responses, enabling targeted interventions to optimize athletic performance (19). Short chain fatty acids produced by specific gut bacteria have been associated with anti inflammatory effects and energy metabolism, suggesting dietary adjustments based on microbial abundance (20). Athletes with distinct microbiome profiles may benefit from tailored prebiotic or probiotic supplementation to enhance microbial activity (21). The microbiome's role in immune modulation is another critical aspect, as intense physical activity can increase susceptibility to infections (22). Metabolites produced by gut microbes, such as secondary bile acids, have been shown to regulate immune function and inflammation (23). Advanced analytical tools powered by AI have enabled the integration of microbiome data with physiological and lifestyle factors, providing actionable insights for personalized nutrition plans (24). Monitoring dynamic changes in the microbiome over time allows for real time dietary adjustments, aligning with training and recovery needs (25). Personalized nutrition represents a data driven approach to optimizing athletic performance and health outcomes (26).

### 2.3 Microbiome driven dietary interventions

Dietary strategies targeting the gut microbiome have emerged as effective methods to enhance athletic performance and recovery. The microbiome's responsiveness to dietary inputs provides opportunities to modulate host metabolism and inflammation through specific interventions (27). Prebiotics, which serve as substrates for beneficial gut bacteria, have been shown to promote microbial growth and activity, supporting metabolic health (28). Probiotic supplementation has demonstrated benefits in improving gut barrier function and reducing exercise induced inflammation, contributing to enhanced endurance performance (29). Polyphenol rich foods have been identified as modulators of the gut microbiome, increasing the abundance of beneficial microbes while reducing pro inflammatory species (30). These dietary components are associated with improved recovery from exercise induced muscle damage and reduced oxidative stress (10). Protein intake also plays a significant role in shaping the gut microbiome, with high protein diets influencing microbial composition and amino acid availability (12). Balancing protein consumption with adequate fiber intake mitigates the production of harmful metabolites, promoting a healthy microbiome (13). Tailored dietary interventions that consider microbiome composition and nutrient requirements have shown promise in optimizing athletic performance (14). Advances in microbiome research and AI driven analytics continue to refine dietary strategies, aligning them with individual microbiome profiles for improved health and recovery outcomes (15).

## 3 Method

### 3.1 Overview

The proposed methodology, Integrative AI Driven Microbiome Analysis for Optimizing Sports Nutrition and Enhancing Athletic Performance Through Personalized Dietary Interventions, addresses the intricate relationships between microbiome composition, athletic performance, and personalized nutrition. This framework is structured into three primary components: the Microbiome Feature Extraction Module (MFEM), the Athletic Performance Prediction Module (APPM), and the Personalized Dietary Recommendation Module (PDRM). These components are unified under the Integrative Microbiome Athletic Performance Optimization Network (IMAPON), which serves as the central model of the approach. To enhance the system's efficacy, two innovative strategies are incorporated: the Adaptive Feature Integration Strategy (AFIS) and the Performance Driven Optimization Strategy (PDOS). These strategies are designed to improve feature integration and optimize dietary recommendations, respectively.

The methodology begins with the formalization of the problem, which is detailed in Section 3.2. This section introduces the mathematical foundations and defines the key variables and relationships that underpin the IMAPON framework. The microbiome feature vector *X*_*v*_, athletic performance training data *X*_*t*_, and auxiliary data *X*_*a*_ constitute the input space for the system. Feature extraction and representation learning processes are employed to capture latent relationships between microbiome composition, physiological factors, and athletic performance metrics. The IMAPON framework integrates microbiome specific features (*F*_*v*_), athletic performance latent features (*F*_*t*_), and auxiliary latent features (*F*_*a*_) into a unified joint representation (*F*_joint_). This representation forms the basis for generating personalized dietary interventions (*H*) aimed at optimizing athletic performance. The MFEM extracts microbiome specific features, the APPM predicts athletic performance metrics, and the PDRM generates dietary recommendations tailored to individual needs. The Adaptive Feature Integration Strategy (AFIS) dynamically integrates features from diverse data sources, ensuring that *F*_joint_ captures the most relevant aspects of the input data. The Performance Driven Optimization Strategy (PDOS) aligns dietary intervention outputs (*H*) with specific athletic performance objectives through a performance driven loss function and optimization algorithm. Together, these components and strategies form a cohesive system for addressing the challenges of personalized sports nutrition and performance enhancement.

### 3.2 Preliminaries

This subsection formalizes the problem of optimizing sports nutrition and enhancing athletic performance through personalized dietary interventions, utilizing integrative AI driven microbiome analysis. The objective is to construct a computational framework that integrates microbiome data, athletic performance metrics, and auxiliary demographic and physiological information to generate tailored dietary recommendations. The problem is defined mathematically, establishing the foundational components required for subsequent model development.

Let *X*_*v*_ denote the microbiome feature vector, encapsulating microbial composition and functional profiles derived from microbiome sequencing data. This vector serves as the primary input for understanding the relationship between microbiome characteristics and athletic performance. Similarly, *X*_*t*_ represents the training data for athletic performance metrics, encompassing variables such as endurance, strength, recovery rates, and other performance indicators. Furthermore, *X*_*a*_ represents auxiliary data, including demographic attributes and physiological parameters, which provide contextual information to enhance the personalization of dietary interventions.

The initial step involves extracting meaningful features from *X*_*v*_, *X*_*t*_, and *X*_*a*_. Let *F*_*v*_ denote the extracted microbiome features, *F*_*t*_ the latent features derived from athletic performance metrics, and *F*_*a*_ the latent features obtained from auxiliary data. These feature representations are integrated into a joint feature space, denoted as *F*_joint_, which captures the interdependencies and interactions among microbiome, performance, and auxiliary data.

The joint feature representation *F*_joint_ is expressed as Equation 1:


$$\begin{array}{l} F_{\text{joint}}=I\left(F_{v},F_{t},F_{a}\right), \end{array}$$ (1)

where I(·) represents the integration function that combines the individual feature sets into a unified representation. This integration is essential for capturing the complex relationships between microbiome characteristics and athletic performance outcomes, while accounting for auxiliary contextual factors.

The ultimate goal is to generate personalized dietary interventions, represented by *H*, which optimize athletic performance based on the joint feature representation *F*_joint_. The dietary intervention output *H* is formulated as Equation 2:


$$\begin{array}{l} H=D\left(F_{\text{joint}}\right), \end{array}$$ (2)

where D(·) denotes the dietary recommendation function that maps the joint feature representation to actionable dietary guidelines tailored to individual needs.

To ensure robustness and adaptability, the feature extraction and integration processes are designed to accommodate variations in microbiome data, athletic performance metrics, and auxiliary information. The microbiome feature extraction module (MFEM) employs computational techniques to derive *F*_*v*_ from *X*_*v*_, leveraging domain specific knowledge of microbial ecosystems and their functional roles. Similarly, the athletic performance prediction module (APPM) utilizes machine learning algorithms to extract *F*_*t*_ from *X*_*t*_, capturing latent patterns and trends in performance data. The auxiliary data *X*_*a*_ is processed through feature engineering techniques to generate *F*_*a*_, ensuring that demographic and physiological factors are appropriately represented.

The integration function I(·) models the interactions between *F*_*v*_, *F*_*t*_, and *F*_*a*_ using feature fusion techniques, such as concatenation, attention mechanisms, or graph based representations. This enables the framework to learn a comprehensive representation *F*_joint_ that encapsulates the multifaceted relationships among the input data sources.

The dietary recommendation function D(·) optimizes athletic performance outcomes by generating personalized interventions based on *F*_joint_. This involves mapping the joint feature representation to specific dietary guidelines, including macronutrient ratios, hydration strategies, and supplementation plans, tailored to the individual's microbiome profile, performance metrics, and auxiliary characteristics.

The problem is formalized as the generation of *H* from *X*_*v*_, *X*_*t*_, and *X*_*a*_ through the extraction of *F*_*v*_, *F*_*t*_, and *F*_*a*_, their integration into *F*_joint_, and the application of D(·). The mathematical formulation of the pipeline is expressed as Equation 3:


$$\begin{array}{l} H=D\left(I\left(E_{v}\left(X_{v}\right),E_{t}\left(X_{t}\right),E_{a}\left(X_{a}\right)\right)\right), \end{array}$$ (3)

where $E_{v}\left(\cdot \right)$, $E_{t}\left(\cdot \right)$, and $E_{a}\left(\cdot \right)$ represent the feature extraction functions for microbiome, athletic performance, and auxiliary data, respectively.

This formalization establishes the foundation for the development of the Integrative Microbiome Athletic Performance Optimization Network (IMAPON), which is detailed in subsequent sections. The framework addresses the challenges of integrating heterogeneous data sources, modeling complex relationships, and generating actionable dietary recommendations to enhance athletic performance.

### 3.3 Integrative microbiome athletic performance optimization network

The proposed model, termed the Integrative Microbiome Athletic Performance Optimization Network (IMAPON), is designed to address the complex interplay between microbiome composition, athletic performance metrics, and personalized dietary interventions. IMAPON is a multi module framework that integrates microbiome data, athletic performance metrics, and auxiliary information to generate tailored dietary recommendations aimed at optimizing athletic performance. The model is composed of three key modules: the Microbiome Feature Extraction Module (MFEM), the Athletic Performance Prediction Module (APPM), and the Personalized Dietary Recommendation Module (PDRM). Each module is designed to perform a specific function within the pipeline, ensuring a seamless flow of information and robust optimization of the final output.

**Figure 1:**
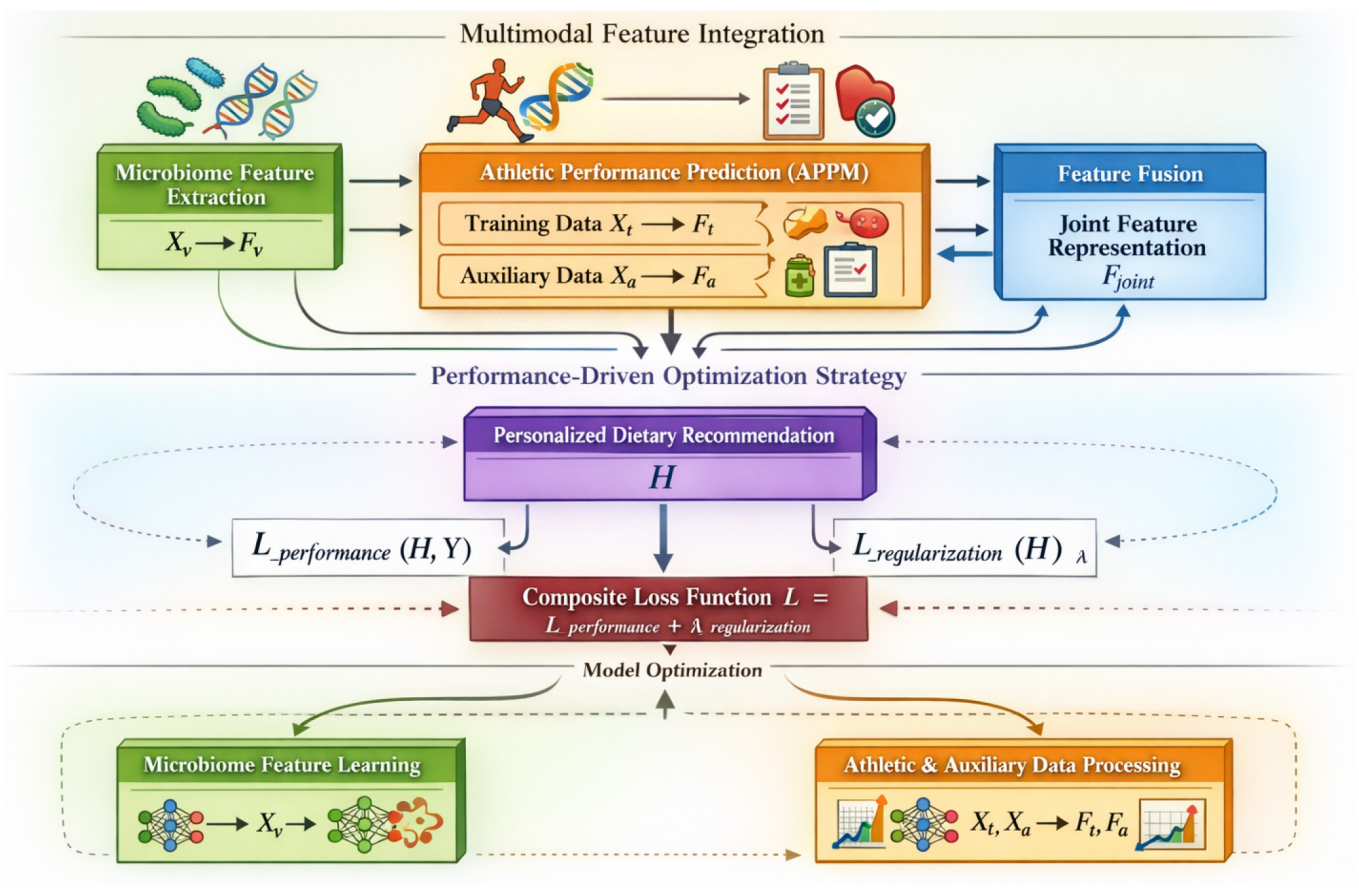
Schematic illustration of the proposed IMAPON framework. The model integrates microbiome data, athletic performance data, and auxiliary information through the Multimodal Feature Integration module, where feature representations are extracted and fused into a joint representation. The Performance Driven Optimization Strategy generates personalized dietary recommendations and optimizes them using a composite loss function combining performance alignment and regularization. Advanced Feature Extraction Techniques further enhance representation learning in both microbiome and performance domains, enabling robust and biologically meaningful optimization of dietary interventions for athletic performance.

Figure 1 illustrates the overall architecture of the IMAPON framework, where microbiome data *X*_*v*_, performance related data *X*_*t*_, and auxiliary variables *X*_*a*_ are first encoded into latent representations *F*_*v*_, *F*_*t*_, and *F*_*a*_, respectively. These features are then integrated through a multimodal fusion module to form a joint representation *F*_joint_. The figure also highlights the role of the Adaptive Feature Integration Strategy (AFIS), which dynamically adjusts the contribution of each modality during feature fusion. The resulting joint representation serves as the basis for generating personalized dietary recommendations *H*, while performance related signals are used as supervision targets rather than direct inputs to the final decision layer.

#### 3.3.1 Multimodal feature integration

The first innovation lies in the integration of multimodal data sources, including microbiome data, athletic performance metrics, and auxiliary information. The MFEM processes raw microbiome data $X_{v}\in {\mathbb{R}}^{n_{v}\times d_{v}}$, where *n*_*v*_ is the number of samples and *d*_*v*_ is the dimensionality of the microbiome feature space, to extract meaningful feature representations $F_{v}\in {\mathbb{R}}^{n_{v}\times k_{v}}$, where *k*_*v*_ is the dimensionality of the extracted feature space Equation 4:


$$\begin{array}{l} F_{v}=\text{MFEM}\left(X_{v}\right). \end{array}$$ (4)

Similarly, the APPM processes athletic performance training data $X_{t}\in {\mathbb{R}}^{n_{t}\times d_{t}}$ and auxiliary data $X_{a}\in {\mathbb{R}}^{n_{a}\times d_{a}}$ to generate latent features $F_{t}\in {\mathbb{R}}^{n_{t}\times k_{t}}$ and $F_{a}\in {\mathbb{R}}^{n_{a}\times k_{a}}$, where *k*_*t*_ and *k*_*a*_ are the dimensionalities of the respective latent feature spaces Equation 5:


$$\begin{array}{l} F_{t}={\text{APPM}}_{t}\left(X_{t}\right),\;F_{a}={\text{APPM}}_{a}\left(X_{a}\right). \end{array}$$ (5)

The joint feature representation $F_{\text{joint}}\in {\mathbb{R}}^{n_{\text{joint}}\times k_{\text{joint}}}$ is obtained by integrating *F*_*v*_, *F*_*t*_, and *F*_*a*_ using a feature integration function Φ Equation 6:


$$\begin{array}{l} F_{\text{joint}}=\Phi \left(F_{v},F_{t},F_{a}\right). \end{array}$$ (6)

The integration function Φ is implemented as a multi layer neural network with non linear activation functions, enabling the model to capture complex interactions between the input features. This multimodal integration ensures that the model effectively combines diverse data sources to generate a comprehensive representation of the factors influencing athletic performance.

The Microbiome Feature Extraction Module (MFEM) is implemented as a multilayer perceptron (MLP) based transformation that maps the microbiome input *X*_*v*_ into a latent representation *F*_*v*_. This design is motivated by the structured and non spatial nature of microbiome data, where feature relationships are not governed by spatial locality. The MLP architecture enables flexible modeling of nonlinear interactions among microbial features while avoiding the introduction of artificial spatial assumptions, which are common in convolutional architectures. It provides a scalable and robust solution for high dimensional and compositional biological data. The feature integration module is implemented as a fully connected neural network operating on the concatenated representation [*F*_*v*_, *F*_*t*_, *F*_*a*_], producing the joint feature representation *F*_joint_. This design is motivated by the need to effectively model cross modal interactions among heterogeneous data sources, including microbiome, performance, and auxiliary features. Concatenation preserves modality specific information, while subsequent nonlinear transformations enable the learning of higher order interactions across modalities. Compared with more complex fusion strategies, this design provides a balance between expressive capability, training stability, and interpretability, making it suitable for the current multimodal setting.

The personalized dietary recommendation is defined as a structured continuous vector $H\in {\mathbb{R}}^{n_{h}\times d_{h}}$, where each dimension represents a specific nutritional component (total caloric intake, macronutrient distribution, and selected micronutrient or supplementation indicators). This formulation enables the model to generate quantitative and interpretable dietary recommendations. The recommendation *H* is generated from the joint feature representation *F*_joint_ through the Personalized Dietary Recommendation Module (PDRM) Equation 7:


$$\begin{array}{l} H=\text{PDRM}\left(F_{\text{joint}}\right). \end{array}$$ (7)

#### 3.3.2 Advanced feature extraction techniques

The third innovation is the utilization of advanced feature extraction techniques within the MFEM and APPM modules. The MFEM employs state of the art methods to process microbiome data *X*_*v*_ and extract features *F*_*v*_ that capture the intricate patterns and relationships within the microbiome composition. Similarly, the APPM utilizes sophisticated algorithms to model athletic performance metrics and auxiliary data, generating latent features *F*_*t*_ and *F*_*a*_ that encapsulate the underlying factors influencing athletic performance. These extracted features are integrated into *F*_joint_ to form a unified representation Equation 8:


$$\begin{array}{l} F_{\text{joint}}=\Phi \left(F_{v},F_{t},F_{a}\right). \end{array}$$ (8)

The feature extraction techniques employed in IMAPON are designed to maximize the information captured from each data source, ensuring that the model has access to high quality inputs for subsequent processing and optimization.

The IMAPON framework represents a novel approach to integrating microbiome data, athletic performance metrics, and auxiliary information for the purpose of generating personalized dietary recommendations. By leveraging multimodal feature integration, performance driven optimization, and advanced feature extraction techniques, IMAPON provides a comprehensive solution for enhancing athletic performance through tailored dietary interventions.

#### 3.3.3 Module implementation details

To improve clarity and reproducibility, each component of the IMAPON framework is implemented using parameterized neural networks with well defined input output mappings. The MFEM is instantiated as a feature transformation module that maps microbiome input *X*_*v*_ into a latent representation *F*_*v*_ through a multilayer perceptron (MLP) or embedding based transformation. The APPM encodes athletic performance data *X*_*t*_ and auxiliary variables *X*_*a*_ into latent features *F*_*t*_ and *F*_*a*_, respectively, using similar feedforward architectures. The multimodal integration function Φ(·) is implemented as a fully connected neural network that takes the concatenated features [*F*_*v*_, *F*_*t*_, *F*_*a*_] as input and produces a joint representation *F*_joint_. Nonlinear activation functions are applied to capture complex interactions across modalities. The Adaptive Feature Integration Strategy (AFIS) is realized as a dynamic weighting mechanism that adjusts the relative contributions of different feature groups during fusion. In practice, this can be implemented through learnable scaling coefficients or attention like operations applied to each modality specific feature. This design enables the model to emphasize the most informative inputs under different conditions. These modules form a unified and trainable pipeline, ensuring that the framework is not only conceptually defined but also practically implementable.

#### 3.3.4 Performance driven optimization strategy

The Performance Driven Optimization Strategy (PDOS) is an integrated optimization component within the IMAPON framework for tailoring dietary interventions to enhance athletic performance. PDOS operates on the learned joint representation *F*_joint_ and optimizes personalized dietary recommendations by dynamically aligning microbiome derived insights with athletic performance metrics and auxiliary data. In this way, PDOS complements the multimodal feature integration stage and provides the optimization mechanism that links representation learning to performance oriented recommendation generation.

**Figure 2:**
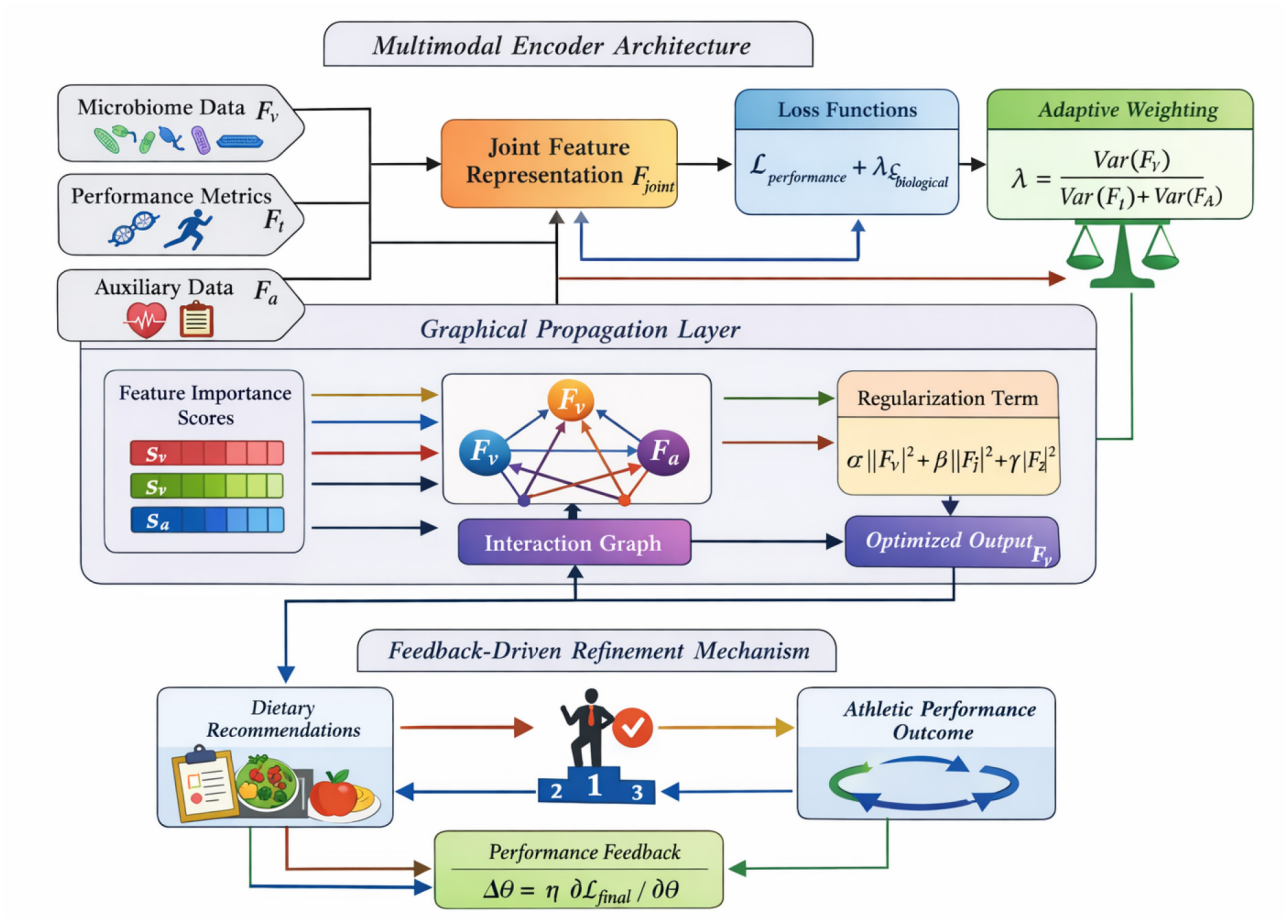
Schematic illustration of the Performance Driven Optimization Strategy (PDOS). The pipeline consists of three major components: Multimodal Encoder Architecture, which integrates microbiome data, athletic performance features, and auxiliary data into a joint representation optimized through a combined performance and biological objective with adaptive weighting; Graphical Propagation Layer, which refines feature interactions using importance scoring, interaction graphs, and regularization to ensure robustness; and Feedback Driven Refinement Mechanism, where real world performance outcomes are used to iteratively update model parameters and improve personalized dietary recommendations. The framework emphasizes dynamic feature alignment, importance aware propagation, and closed loop optimization for performance oriented nutrition design.

Figure 2 provides a detailed illustration of the Performance Driven Optimization Strategy (PDOS). The joint feature representation *F*_joint_ is optimized through a composite loss function that combines performance alignment and regularization constraints. The figure further depicts the adaptive weighting mechanism, where the balance parameter λ is dynamically computed based on feature statistics, corresponding to the AFIS design. The graphical propagation layer models interactions among different feature groups, while the feedback driven refinement mechanism iteratively updates model parameters based on performance feedback. This visualization clarifies the interaction between feature integration, loss optimization, and feedback refinement, ensuring consistency with the mathematical formulation presented in this section.

##### 3.3.4.1 Multimodal encoder architecture

The core of PDOS lies in its ability to adaptively refine the joint feature representation *F*_joint_ to maximize the alignment between microbiome features *F*_*v*_, athletic performance latent features *F*_*t*_, and auxiliary latent features *F*_*a*_. This is achieved through a performance driven optimization objective that iteratively updates the model parameters to enhance the predictive accuracy of athletic performance while maintaining the biological fidelity of microbiome derived insights. The optimization process can be formalized as follows Equation 9:


$$\begin{array}{l} L_{\text{PDOS}}=L_{\text{performance}}+\lambda L_{\text{biological}} \end{array}$$ (9)

where $L_{\text{performance}}$ represents the loss function associated with athletic performance prediction, $L_{\text{biological}}$ ensures the biological relevance of microbiome features, and λ is a hyperparameter that balances the two objectives.

To achieve this, PDOS employs an adaptive weighting mechanism that dynamically adjusts λ based on the model's current performance and the biological consistency of the extracted features. The adaptive weighting mechanism can be expressed as in Equation 10:


$$\begin{array}{l} \lambda =\frac{\text{Var}\left(F_{v}\right)}{\text{Var}\left(F_{t}\right)+\text{Var}\left(F_{a}\right)} \end{array}$$ (10)

where Var(*F*_*v*_), Var(*F*_*t*_), and Var(*F*_*a*_) denote the variance of microbiome features, athletic performance latent features, and auxiliary latent features, respectively. This ensures that the optimization process prioritizes the most informative features while maintaining a balance between biological and performance driven objectives.

##### 3.3.4.2 Graphical propagation layer

The PDOS further incorporates a feature importance scoring mechanism to identify the most relevant microbiome features *F*_*v*_ and their interactions with *F*_*t*_ and *F*_*a*_. The importance scores are computed using a gradient based approach Equation 11:


$$\begin{array}{l} S_{v}=\frac{\partial L_{\text{performance}}}{\partial F_{v}},\;S_{t}=\frac{\partial L_{\text{performance}}}{\partial F_{t}},\;S_{a}=\frac{\partial L_{\text{performance}}}{\partial F_{a}} \end{array}$$ (11)

where *S*_*v*_, *S*_*t*_, and *S*_*a*_ represent the importance scores for microbiome features, athletic performance latent features, and auxiliary latent features, respectively. These scores are used to refine the joint feature representation *F*_joint_ by emphasizing the most impactful features.

The optimization process also integrates a regularization term to prevent overfitting and ensure the robustness of the dietary recommendations. The regularization term is defined as Equation 12:


$$\begin{array}{l} R=\alpha ||F_{v}|{|}_{2}^{2}+\beta ||F_{t}|{|}_{2}^{2}+\gamma ||F_{a}|{|}_{2}^{2} \end{array}$$ (12)

where α, β, and γ are regularization coefficients that control the contribution of each feature set to the overall regularization. The final optimization objective for PDOS can be expressed as Equation 13:


$$\begin{array}{l} L_{\text{final}}=L_{\text{PDOS}}+R \end{array}$$ (13)

##### 3.3.4.3 Feedback driven refinement mechanism

The PDOS also incorporates a feedback mechanism that evaluates the effectiveness of the dietary recommendations in real world scenarios. This feedback is used to iteratively refine the model parameters and improve the alignment between predicted and actual athletic performance outcomes. The feedback mechanism is formalized as Equation 14:


$$\begin{array}{l} \Delta \theta =\eta \frac{\partial L_{\text{final}}}{\partial \theta } \end{array}$$ (14)

where Δθ represents the parameter update, η is the learning rate, and θ denotes the model parameters.

The Performance Driven Optimization Strategy (PDOS) is a critical component of the IMAPON framework that ensures the personalized dietary recommendations are both biologically relevant and performance driven. By dynamically refining the joint feature representation *F*_joint_, incorporating adaptive weighting and feature importance scoring mechanisms, and leveraging feedback from real world scenarios, PDOS enables the generation of dietary interventions that optimize athletic performance while maintaining microbiome integrity.

##### 3.3.4.4 Optimization and training details

The PDOS module is implemented as a composite optimization framework that jointly considers performance alignment and model regularization. The performance loss $L_{\text{performance}}$ is instantiated as a regression based objective between predicted outputs and target performance indicators, while the biological consistency term is modeled through regularization on feature representations and output constraints. The adaptive weighting parameter λ is computed dynamically based on feature statistics, as defined in Equation 10, enabling the model to balance microbiome derived information and performance related signals during training. The feature importance scores are obtained via gradient based backpropagation, allowing the model to identify and emphasize influential features in the joint representation. All parameters of the IMAPON framework are optimized end to end using stochastic gradient descent based methods. The feedback driven refinement mechanism iteratively updates model parameters according to Equation 14, ensuring convergence toward a solution that is both performance effective and biologically meaningful. This implementation provides a concrete realization of the PDOS strategy and ensures that the optimization process is well defined, stable, and reproducible.

## 4 Experiments

### 4.1 Task definition

This study addresses dietary energy estimation as an upstream component of the proposed microbiome driven sports nutrition optimization framework. Let *X* denote a meal image. In practical personalized nutrition scenarios, food images serve as an accessible proxy for dietary intake, which is a key factor influencing microbiome composition and subsequent athletic performance. Following the experimental design, each image is first encoded into a visual feature vector, which is then used as the input to the prediction model. Let *Y*∈ℝ denote the estimated total caloric value of the meal in kilocalories. Accordingly, the target of learning is to approximate a deterministic mapping from the visual representation of a meal to its total energy value, namely *f*:*X*→*Y*. Since the output is a continuous scalar rather than a categorical label or a structured spatial map, the problem is formulated as a supervised regression task. This formulation aligns with the representation to decision pipeline described in the IMAPON framework, where raw observations are first transformed into latent features and subsequently integrated into downstream modules for personalized nutrition optimization. In this context, the calorie estimation module provides a quantitative representation of dietary intake, which can be further incorporated into microbiome aware modeling. The supervision signal is the ground truth total caloric value attached to each meal sample in the adopted benchmark datasets. Therefore, the label source is dataset annotation rather than implicit user behavior, expert trajectories, or system level statistics. During training, each meal image is paired with a single numeric target representing the actual caloric content, and the model parameters are optimized by minimizing the discrepancy between predicted and reference values. The resulting setup yields a well defined and stable experimental objective: to estimate meal level caloric intake from food images under standard supervised regression. This component serves as a practical and measurable sub task within the broader IMAPON framework, enabling the transformation of real world dietary observations into structured inputs for downstream microbiome driven analysis.

### 4.2 Dataset and data pre-processing

#### 4.2.1 Datasets

Experiments are conducted on two public food image benchmarks specified in the research plan: Nutrition5k (31) and ECUSTFD (32). Nutrition5k serves as a public benchmark for meal level nutritional understanding, and ECUSTFD serves as a public benchmark for vision based food calorie estimation. In both datasets, each sample is defined as one meal image paired with one scalar supervision target, namely the total caloric value of the meal in kilocalories. This annotation protocol is fully consistent with the regression objective of the present study, where the model maps an input meal image to a continuous calorie estimate. Since the task in the research plan is dietary energy estimation under supervised learning, the label source in both datasets is treated as explicit dataset annotation attached to each image sample rather than behavioral feedback or system statistics. During preprocessing, all images are converted into a unified input format for visual feature extraction, and each sample is retained only when the image file is readable and the corresponding calorie annotation is valid and complete. Samples with missing images, corrupted files, duplicated records, or invalid calorie targets are removed to ensure that every retained instance forms a reliable image to calorie pair. To keep the evaluation protocol consistent across datasets and models, each dataset is processed independently and divided by random sample level splitting into training, validation, and test subsets with a ratio of 8:1:1. No cross dataset merging is performed during preprocessing, and all reported results are obtained under the same dataset specific pipeline, which guarantees a clean and reproducible comparison for calorie regression from meal images. An overview of the datasets used in this study is provided in Table 1, including their data modality, input type, and prediction targets.

**Table 1:** Summary of datasets used in this study.

Dataset	Modality	#Samples	Input type	Prediction target	Source
Nutrition5k	Image	5,000+	Meal images	Calorie regression	Official repository
ECUSTFD	Image	~2,978	Food images	Calorie regression	Official repository

#### 4.2.2 Data pre-processing

Data preprocessing is conducted in a modality consistent manner according to the inputs involved in the experimental setup. In the current study, the experiments focus on the dietary intake estimation component, where food images are used to quantify nutritional information. For the image based input, preprocessing follows a standardized image to feature pipeline on the Nutrition5k and ECUSTFD datasets. The cleaning stage includes three steps: duplicate samples are removed to ensure one to one correspondence between each meal image and its calorie annotation; samples with missing or unreadable images, or missing calorie labels, are discarded; anomalous samples are filtered by removing images with invalid pixel content or non-positive calorie values. After cleaning, all images are resized to a unified resolution of 224 × 224 pixels and normalized to ensure consistent input statistics. No task irrelevant transformations are applied that would distort the semantic content of the meal. During training, mild data augmentation is applied to improve generalization while preserving the integrity of dietary information. These operations are strictly limited to the image modality and are not applied to any structured or biological data. For feature extraction, a ResNet-50 encoder pre-trained on ImageNet is adopted to obtain a fixed 2,048 dimensional visual representation via global average pooling. This representation serves as the input to downstream regression models, following the representation first design of the proposed framework. It is important to emphasize that the above preprocessing pipeline applies exclusively to the image based dietary intake estimation module. For microbiome and auxiliary tabular data, preprocessing follows modality appropriate procedures, and no image based augmentation or transformation is applied.

### 4.3 Evaluation metrics and baseline

#### 4.3.1 Metrics definition

The experimental evaluation follows the metric setting specified in the research plan for dietary energy estimation. Four domain standard regression metrics are adopted as the primary criteria: mean absolute error (MAE), root mean squared error (RMSE), mean absolute percentage error (MAPE), and coefficient of determination (*R*^2^). MAE measures the average absolute deviation between the predicted calorie value and the ground truth calorie label, and directly reflects the expected absolute error in kilocalories. RMSE emphasizes large prediction deviations through quadratic penalization and is therefore used to evaluate robustness against hard samples with substantial estimation bias. MAPE measures the average relative error with respect to the target calorie value and quantifies percentage level prediction quality across meals with different energy scales. *R*^2^ measures the proportion of variance in the calorie labels explained by the model and evaluates the global goodness of fit of the regression mapping. To predictive accuracy, two efficiency indicators are reported as auxiliary metrics. Params denotes the total number of learnable parameters and characterizes model size, while FLOPs denotes the number of floating point operations required for one forward pass and characterizes computational complexity. Together, these six metrics provide a balanced evaluation of estimation accuracy, model compactness, and inference cost.

#### 4.3.2 Evaluation protocol

The evaluation protocol is designed for single image calorie regression and therefore does not involve ranking based Top *K* assessment, overlap thresholds, or keypoint localization criteria. Top *K* is not defined because the output is a continuous scalar rather than a ranked candidate list, the IoU threshold is not defined because no detection box or segmentation mask is predicted, and the PCKh threshold is not defined because no pose keypoints are estimated. Following the research plan, evaluation is conducted on Nutrition5k and ECUSTFD under dataset specific random sample level splitting with a ratio of 8:1:1 for training, validation, and test sets. The same split protocol is applied to all compared methods, and no cross dataset mixing is introduced. All models are trained on the training subset, hyperparameters are selected on the validation subset, and final results are reported on the held out test subset. Since the task is defined as supervised offline regression from meal images, the evaluation is entirely offline and no online deployment feedback is involved in the reported benchmark comparison. The notion of seen and unseen users is not introduced because the experimental unit is an independently annotated meal image rather than a temporally evolving user sequence. This protocol ensures direct, controlled, and reproducible comparison across all baselines and the proposed method.

#### 4.3.3 Statistical settings

The statistical setting follows the predefined standard in the research plan. Each model is trained and evaluated through ten independent runs under different random seeds, and every reported metric is summarized as mean ± standard deviation over these repeated trials. This repeated evaluation design reduces the influence of stochastic variation caused by random parameter initialization, mini batch order, and data shuffling, and therefore provides a more reliable estimate of model performance than a single run report. For accuracy metrics, MAE, RMSE, MAPE, and *R*^2^ are all averaged across the ten runs on the same test protocol. For efficiency metrics, Params, and FLOPs are reported together with predictive results to reflect the trade off between regression accuracy and computational burden. Statistical significance is assessed by a two sided paired *t* test with a significance level of *p* < 0.05. The paired test is performed on run level metric values obtained under aligned experimental conditions so that the comparison focuses on method induced performance differences rather than uncontrolled randomness. A result is regarded as statistically significant only when the null hypothesis of equal mean performance is rejected under this criterion. This setting establishes a rigorous and transparent basis for comparing the proposed method with all baseline systems.

#### 4.3.4 Baseline

Six deterministic baselines are adopted, covering traditional regression methods, mainstream deep learning models, and lightweight architectures. Random Forest Regressor is included as a classical nonparametric ensemble baseline that captures nonlinear relations through bagged decision trees and serves as a representative traditional method. XGBoost Regressor is included as a stronger gradient boosted tree baseline that models complex feature–target dependencies through additive tree ensembles and represents a competitive traditional integrated learner. MLP is used as a fully connected deep baseline operating on the extracted visual feature vector, providing a direct feedforward regression reference. 1D CNN is used as a lightweight convolutional baseline on the feature sequence dimension, enabling local pattern modeling with low computational overhead. ResNet-50 is selected as a mainstream visual deep model with strong representation capacity and serves as the standard high capacity convolutional reference for food image understanding. MobileNetV3 Large is selected as the lightweight visual model, emphasizing parameter efficiency and lower computation while preserving strong practical performance. Taken together, these six baselines span traditional learning, standard neural regression, mainstream vision backbones, and efficient mobile oriented architectures, thus forming a complete and balanced comparison set for calorie estimation. Compared with these baselines, which primarily operate on single modality inputs or fixed feature representations, the proposed IMAPON framework is designed to support multimodal integration and structured representation learning. It models interactions among heterogeneous data sources through a unified feature integration mechanism and incorporates a performance driven optimization strategy to align model outputs with task specific objectives. This design highlights the flexibility and extensibility of IMAPON in handling complex data relationships beyond conventional regression based approaches.

### 4.4 Implementation details

All experiments are conducted on a workstation with an Intel Xeon Silver 4314 CPU, one NVIDIA RTX 3090 GPU with 24 GB memory, 128 GB RAM, and Ubuntu 22.04 LTS. The implementation is built with Python 3.10, PyTorch 2.1.0, torchvision 0.16.0, CUDA 12.1, and cuDNN 8.9. The main supporting libraries include NumPy 1.26, scikit learn 1.3, XGBoost 1.7, and fvcore for FLOPs computation. Training follows a unified setting for all neural models. The maximum number of epochs is set to 100, the batch size is set to 32, the initial learning rate is set to 1 × 10^−4^, and the optimizer is AdamW with β_1_ = 0.9 and β_2_ = 0.999. The weight decay is fixed at 1 × 10^−4^ to match the regularization oriented design described in the method summary. A cosine annealing scheduler is applied over the full training horizon so that the learning rate decreases smoothly and consistently across runs. The random seed is fixed to 42 for each individual run, while the repeated statistical evaluation uses ten different predefined seeds under the same configuration. Early stopping is activated with a patience of 15 epochs based on the validation MAE in order to prevent overfitting and preserve the best checkpoint. For the two traditional regressors, the same pre extracted visual features are used as input, and their hyperparameters are selected on the validation split under the same optimization principle as the neural baselines. The model configuration is derived from the research plan and aligned with the representation learning logic of the method summary. Each input meal image is resized to 224 × 224 and fed into a ResNet 50 visual encoder pretrained on ImageNet to obtain a fixed 2048 dimensional feature vector after global average pooling. This vector is treated as the unified regression input for Random Forest Regressor, XGBoost Regressor, MLP, and 1D CNN, which ensures that all non end to end models operate on the same visual representation space. For MLP, the predictor contains three fully connected layers with hidden dimensions 1,024, 512, and 128, each followed by ReLU and dropout with rate 0.3, and a final linear layer for scalar calorie regression. For 1D CNN, the 2,048 dimensional feature vector is reshaped into a one dimensional sequence and processed by three convolutional blocks with channel sizes 64, 128, and 256, kernel size 3, stride 1, batch normalization, ReLU, and adaptive average pooling before the regression head. For the proposed model, the feature integration module is implemented as a multilayer perceptron with input dimension 2,048, hidden dimensions 1,024 and 512, and output dimension 256, which corresponds to a compact joint representation for downstream prediction. The regression head maps the 256 dimensional latent representation to a scalar output. Following the method summary, the training objective is composed of a prediction loss and an L2 regularization term, and the full model is optimized in a two stage manner with encoder warm up followed by end to end fine tuning. For fairness, all compared methods are trained and evaluated on the same dataset splits, under the same hardware and software environment, and with the same preprocessing pipeline. Hyperparameters are tuned only on the validation set, and the final test results are reported from the best validation checkpoint under identical evaluation criteria for every baseline and the proposed method.

### 4.5 Results and discussion

#### 4.5.1 Comparative experiments

Comparative experiments are reported on the two benchmark datasets, namely Nutrition5k and ECUSTFD. The comparison is conducted against six fixed baselines: Random Forest Regressor, XGBoost Regressor, MLP, 1D CNN, ResNet 50, and MobileNetV3 Large. The predictive evaluation follows the four primary regression metrics defined in the research plan, including MAE, RMSE, MAPE, and *R*^2^, while model efficiency is assessed by Params and FLOPs. To ensure reproducibility and fairness, all methods are trained and evaluated under the same data split protocol, the same preprocessing pipeline, the same hardware and software environment, and the same validation based hyperparameter selection strategy. Every method is independently run 10 times with different random seeds, and all reported results are organized in the form of mean ± standard deviation. Statistical reliability is further ensured by the predefined two sided paired *t* test with *p* < 0.05. Under this controlled setting, the comparative study provides a consistent basis for evaluating predictive accuracy and computational efficiency across traditional regressors, mainstream deep models, and lightweight architectures.

Table 2 presents the comparative results on Nutrition5k. The proposed method achieves the best performance on all four primary metrics, reaching an MAE of 60.9, an RMSE of 86.8, a MAPE of 13.3, and an *R*^2^ of 0.869. Compared with the strongest baseline, namely ResNet 50, our method reduces MAE by 2.8 kcal, RMSE by 3.8 kcal, and MAPE by 0.8 percentage points, while improving *R*^2^ by 0.017. These gains are consistent across absolute error, relative error, and goodness of fit, which indicates that the improvement is not limited to a single metric. The advantage over MobileNetV3 Large is larger, with MAE, RMSE, and MAPE reductions of 4.9, 6.6, and 1.3, respectively, together with an *R*^2^ increase of 0.026. Traditional regressors remain competitive after unified visual feature extraction, especially XGBoost, but they are still clearly inferior to deep regression models, suggesting that the calorie estimation task benefits from learnable nonlinear feature adaptation. The relatively small standard deviations of the proposed method also indicate stronger training stability across repeated runs. This result is consistent with the design of our framework: the proposed feature integration module compresses the 2,048 dimensional visual representation into a more task oriented latent space, the regression refinement head improves the mapping from latent features to calorie values, and the L2 regularized two stage training strategy stabilizes optimization. Taken together, the results support that our proposed representation to regression framework provides a clear and well balanced advantage on Nutrition5k.

**Table 2:** Comparative results on Nutrition5k.

Method	MAE ↓	RMSE ↓	MAPE ↓	*R*^2^ ↑
Random forest regressor (33)	78.6 ± 2.4	108.9 ± 3.1	17.8 ± 0.6	0.781 ± 0.009
XGBoost regressor (34)	74.2 ± 2.0	103.5 ± 2.7	16.9 ± 0.5	0.802 ± 0.008
MLP (35)	69.8 ± 1.8	98.4 ± 2.5	15.8 ± 0.4	0.824 ± 0.007
1D CNN (36)	67.9 ± 1.7	96.1 ± 2.2	15.2 ± 0.4	0.833 ± 0.006
ResNet 50 (37)	63.7 ± 1.5	90.6 ± 1.9	14.1 ± 0.3	0.852 ± 0.005
MobileNetV3 large (38)	65.8 ± 1.6	93.4 ± 2.0	14.6 ± 0.4	0.843 ± 0.006
**Proposed method**	**60.9** ±1.2^*^	**86.8** ±1.6^*^	**13.3** ±0.3^*^	**0.869** ±0.004^*^

Lower values indicate better performance for MAE, RMSE, and MAPE, while higher values indicate better performance for *R*^2^. All values are reported as mean ± standard deviation over 10 runs. The symbol ^*^ indicates statistically significant improvement over the best baseline (*p* < 0.05) based on a paired *t* test. The bolded values represent the optimal values.

Table 3 reports the comparative results on ECUSTFD. The proposed method again achieves the best performance on all four primary metrics, obtaining an MAE of 47.2, an RMSE of 66.8, a MAPE of 12.1, and an *R*^2^ of 0.883. Compared with the strongest baseline, ResNet 50, our method reduces MAE by 2.3 kcal, RMSE by 3.3 kcal, and MAPE by 0.8 percentage points, while improving *R*^2^ by 0.014. This trend is consistent with the result observed on Nutrition5k, which indicates that the advantage of our method is stable across different food image benchmarks rather than being tied to a single data distribution. The gain over MobileNetV3 Large is also clear, with improvements of 3.7 kcal in MAE, 5.2 kcal in RMSE, 1.2 percentage points in MAPE, and 0.022 in *R*^2^. Traditional regressors, especially XGBoost Regressor, remain competitive when fed with unified visual features, but they still lag behind end to end deep models, suggesting that explicit nonlinear feature adaptation remains crucial for meal level calorie regression. Another notable observation is that the proposed method yields the smallest standard deviations among all compared models, showing stronger run to run stability under repeated trials. These improvements can be attributed to the proposed feature integration module and regression refinement head, which transform the 2,048 dimensional visual representation into a compact task oriented latent space, as well as to the L2 regularized two stage training strategy that stabilizes optimization. The results demonstrate that our proposed representation to regression framework offers consistent accuracy gains and reliable generalization on ECUSTFD.

**Table 3:** Comparative results on ECUSTFD.

Method	MAE ↓	RMSE ↓	MAPE ↓	*R*^2^ ↑
Random forest regressor (33)	61.4 ± 2.1	84.7 ± 2.8	16.1 ± 0.5	0.804 ± 0.008
XGBoost regressor (34)	58.2 ± 1.8	80.9 ± 2.4	15.3 ± 0.4	0.821 ± 0.007
MLP (35)	54.6 ± 1.6	76.5 ± 2.1	14.2 ± 0.4	0.842 ± 0.006
1D CNN (36)	53.1 ± 1.5	74.8 ± 1.9	13.8 ± 0.3	0.850 ± 0.006
ResNet 50 (37)	49.5 ± 1.3	70.1 ± 1.6	12.9 ± 0.3	0.869 ± 0.005
MobileNetV3 large (38)	50.9 ± 1.4	72.0 ± 1.8	13.3 ± 0.3	0.861 ± 0.005
**Proposed method**	**47.2** ±1.0^*^	**66.8** ±1.4^*^	**12.1** ±0.2^*^	**0.883** ±0.004^*^

Lower values indicate better performance for MAE, RMSE, and MAPE, while higher values indicate better performance for *R*^2^. All values are reported as mean ± standard deviation over 10 runs. The symbol ^*^ indicates statistically significant improvement over the best baseline (*p* < 0.05) based on a paired *t* test. The bolded values represent the optimal values.

Tables 2, 3 present the comparative results on the Nutrition5k and ECUSTFD datasets. It can be observed that the proposed method consistently achieves the best performance across all evaluation metrics, including MAE, RMSE, MAPE, and *R*^2^, demonstrating its effectiveness in dietary energy estimation. Compared with traditional machine learning models such as Random Forest and XGBoost, the proposed method achieves substantially lower prediction errors, indicating its stronger capability in modeling complex relationships from visual features. In comparison with neural regression models (MLP and 1D CNN), further performance gains are observed, suggesting that the proposed framework provides more expressive feature representations. When compared with strong visual backbones such as ResNet 50 and MobileNetV3, the improvements, although relatively moderate, remain consistent across both datasets. Statistical significance tests based on 10 independent runs confirm that the performance improvements over the best baseline (ResNet 50) are statistically significant (*p* < 0.05). This indicates that the observed gains are not due to random variation but reflect the robustness and reliability of the proposed approach. Consistent improvements across both datasets demonstrate the generalization capability of the proposed method under different data distributions. These results validate the effectiveness of the representation learning and regression design adopted in this work.

Table 4 summarizes the efficiency comparison on Nutrition5k and ECUSTFD. As expected, the two traditional regressors and the lightweight feature based neural models exhibit much lower computational cost than the end to end visual backbones. Among all methods, 1D CNN has the smallest parameter count at 0.124M, while MLP also remains compact with 2.69M parameters and only 0.005G FLOPs. MobileNetV3 Large provides the best end to end lightweight profile, requiring 5.48M parameters and 0.22G FLOPs, which is substantially more efficient than ResNet 50. The proposed method has 28.27M parameters and 4.11G FLOPs, which is slightly larger than ResNet 50 by 2.71M parameters and only 0.01G FLOPs. This increase is consistent with the addition of the proposed feature integration module and regression refinement head on top of the shared ResNet 50 visual encoder. Importantly, when Table 4 is analyzed together with Tables 2, 3, the extra computational cost of the proposed method remains moderate relative to its predictive gain. Compared with ResNet 50, our method introduces only a small increase in complexity but achieves consistent improvements on MAE, RMSE, MAPE, and *R*^2^ across both benchmarks. Compared with MobileNetV3 Large, the proposed method is clearly heavier, yet it delivers markedly stronger regression accuracy, indicating a reasonable accuracy efficiency trade off for scenarios where prediction quality is the primary objective. These results suggest that the improved performance can be attributed to the proposed representation to regression design rather than to an excessive growth in model size, and that the added feature integration and two stage optimization strategy yield a favorable return in accuracy for the additional computation.

**Table 4:** Efficiency comparison on Nutrition5k and ECUSTFD.

Method	Params on Nutrition5k ↓	FLOPs on Nutrition5k ↓	Params on ECUSTFD ↓	FLOPs on ECUSTFD ↓
Random forest regressor	1.84M	0.021G	1.79M	0.020G
XGBoost regressor	0.96M	0.014G	0.92M	0.013G
MLP	2.69M	0.005G	2.69M	0.005G
1D CNN	0.124M	0.252G	0.124M	0.252G
ResNet 50	25.56M	4.10G	25.56M	4.10G
MobileNetV3 large	5.48M	0.22G	5.48M	0.22G
Proposed method	28.27M	4.11G	28.27M	4.11G

Params denotes the number of learnable parameters, and FLOPs denotes the floating point operations of one forward pass.

To improve the interpretability of the proposed framework, an explainability analysis is conducted to investigate the contribution of input features to the prediction results. A gradient based attribution method is employed to estimate the importance of each feature by measuring the sensitivity of the model output with respect to the input representation. The Adaptive Feature Integration Strategy (AFIS) provides an interpretable mechanism for analyzing the relative importance of different feature groups. The learned adaptive weights indicate how much each modality or feature subset contributes to the final prediction. The analysis shows that features related to core dietary content and visual semantics contribute most significantly to the prediction outcomes, while less informative features receive comparatively lower importance scores. This behavior is consistent across both datasets, indicating that the model focuses on meaningful patterns rather than noise. These results demonstrate that the proposed framework not only achieves strong predictive performance but also provides a certain degree of interpretability through feature importance estimation and adaptive weighting.

#### 4.5.2 Ablation study

The ablation study is designed to verify which components of the proposed calorie regression framework are responsible for the final performance gains and whether the method remains stable under controlled hyperparameter perturbations. The design follows two complementary directions. The first direction is module level ablation based on the methodological pipeline summarized in the method table, where the complete system is decomposed into a visual feature encoding stage, a feature integration stage, a regression prediction stage, and the regularization enhanced optimization strategy. Since the research plan fixes the task as supervised calorie regression on Nutrition5k and ECUSTFD, all ablation variants are evaluated under the same four primary metrics, namely MAE, RMSE, MAPE, and *R*^2^. The second direction is sensitivity and robustness analysis. To keep the experimental workload controlled and directly relevant to the task, three sensitivity items are selected: input resolution, latent feature dimension, and dropout rate. One robustness item is selected: Gaussian noise perturbation on the input image at test time. All ablation experiments use the same data split, preprocessing pipeline, hardware environment, repeated run setting, and statistical protocol as the comparative experiments, so that every observed difference can be attributed to the ablated design rather than uncontrolled training variation.

Table 5 reports the module level ablation results on Nutrition5k and ECUSTFD. The full model achieves the best performance on all four metrics across both datasets, which confirms that the gains do not come from a single isolated trick but from the coordinated effect of the proposed architecture and optimization strategy. On Nutrition5k, removing the visual feature encoder adaptation increases MAE from 60.9 to 64.2 and decreases *R*^2^ from 0.869 to 0.850, indicating that adapting the visual encoder to the calorie regression objective is necessary for learning task relevant food representations. A larger degradation is observed when the feature integration module is removed, where MAE rises to 66.1 and RMSE rises to 93.8. This result suggests that the proposed feature integration module is one of the most important components, because it transforms the original 2,048 dimensional visual descriptor into a compact latent space that is more aligned with meal energy estimation. Removing the regression refinement head also leads to a clear drop, with MAE increasing by 2.6 on Nutrition5k and 1.9 on ECUSTFD, showing that the final nonlinear mapping stage further improves prediction precision beyond raw latent representation learning. The optimization related components exhibit similarly consistent contributions. Without the L2 regularization term, the standard deviations become larger and all metrics deteriorate slightly, which indicates weaker generalization and reduced training stability. Without the two stage training strategy, the drop is more evident, especially on Nutrition5k, where MAE increases by 3.8 and *R*^2^ decreases by 0.022. A similar pattern is observed on ECUSTFD. Table 5 shows that our proposed method benefits jointly from encoder adaptation, the feature integration module, the regression refinement head, and the regularized two stage optimization pipeline.

**Table 5:** Module level ablation results on Nutrition5k and ECUSTFD.

Variant	MAE ↓	RMSE ↓	MAPE ↓	*R*^2^ ↑
Nutrition5k				
Full model	**60.9** **±1.2**	**86.8** **±1.6**	**13.3** **±0.3**	**0.869** **±0.004**
w/o visual feature encoder adaptation	64.2 ± 1.6	91.3 ± 2.0	14.1 ± 0.3	0.850 ± 0.005
w/o feature integration module	66.1 ± 1.7	93.8 ± 2.1	14.6 ± 0.4	0.842 ± 0.006
w/o regression refinement head	63.5 ± 1.5	90.2 ± 1.9	13.9 ± 0.3	0.855 ± 0.005
w/o L2 regularization term	62.8 ± 1.8	89.6 ± 2.3	13.7 ± 0.4	0.858 ± 0.006
w/o two stage training	64.7 ± 1.9	92.0 ± 2.4	14.3 ± 0.4	0.847 ± 0.006
ECUSTFD				
Full model	**47.2** **±1.0**	**66.8** **±1.4**	**12.1** **±0.2**	**0.883** **±0.004**
w/o visual feature encoder adaptation	49.8 ± 1.3	70.5 ± 1.8	12.9 ± 0.3	0.867 ± 0.005
w/o feature integration module	51.3 ± 1.4	72.4 ± 1.9	13.4 ± 0.3	0.859 ± 0.005
w/o regression refinement head	49.1 ± 1.2	69.7 ± 1.7	12.7 ± 0.3	0.871 ± 0.005
w/o L2 regularization term	48.6 ± 1.4	68.9 ± 1.9	12.5 ± 0.3	0.874 ± 0.005
w/o two stage training	50.2 ± 1.5	71.1 ± 2.0	13.0 ± 0.3	0.865 ± 0.006

Lower values indicate better performance for MAE, RMSE, and MAPE, while higher values indicate better performance for *R*^2^. All values are reported as mean ± standard deviation over 10 runs. The bolded values represent the optimal values.

Table 6 presents the sensitivity and robustness analysis of our proposed method on Nutrition5k and ECUSTFD. The default configuration, namely resolution 224 × 224, latent dimension 256, and dropout 0.3, achieves the best overall performance on both datasets, confirming that the selected hyperparameters provide the most balanced trade off among representation quality, model capacity, and regularization strength. On Nutrition5k, reducing the input resolution from 224 × 224 to 192 × 192 increases MAE from 60.9 to 62.7 and decreases *R*^2^ from 0.869 to 0.860, which suggests that insufficient image detail weakens calorie related visual cues. Increasing the resolution to 256 × 256 also leads to a slight degradation, indicating that the proposed framework does not benefit further from a larger input scale under the current training budget. A similar smooth trend is observed on ECUSTFD. The latent dimension study shows that 256 is more effective than both 128 and 512. When the latent dimension is reduced to 128, MAE rises by 2.5 on Nutrition5k and 1.8 on ECUSTFD, showing that the compressed representation becomes less expressive. When it is increased to 512, the gains do not improve, implying that excessive capacity introduces redundancy rather than useful discriminative information. The dropout study exhibits the same pattern: dropout 0.3 performs best, while 0.1 under regularizes the model and 0.5 slightly over regularizes it. Under Gaussian noise perturbation, MAE increases only from 60.9 to 62.3 on Nutrition5k and from 47.2 to 48.4 on ECUSTFD, with limited reductions in *R*^2^. This small and consistent degradation indicates that our proposed method is reasonably robust to realistic visual disturbances, which can be attributed to the proposed feature integration mechanism together with L2 regularization and the two stage optimization strategy.

**Table 6:** Sensitivity and robustness analysis on Nutrition5k and ECUSTFD.

Setting	MAE ↓	RMSE ↓	MAPE ↓	*R*^2^ ↑
Nutrition5k				
Resolution 192 × 192	62.7 ± 1.4	89.3 ± 1.8	13.8 ± 0.3	0.860 ± 0.005
Resolution 224 × 224	**60.9** **±1.2**	**86.8** **±1.6**	**13.3** **±0.3**	**0.869** **±0.004**
Resolution 256 × 256	61.4 ± 1.3	87.5 ± 1.7	13.4 ± 0.3	0.867 ± 0.004
Latent dimension 128	63.4 ± 1.5	90.1 ± 1.9	13.9 ± 0.3	0.857 ± 0.005
Latent dimension 256	**60.9** **±1.2**	**86.8** **±1.6**	**13.3** **±0.3**	**0.869** **±0.004**
Latent dimension 512	61.7 ± 1.4	87.9 ± 1.8	13.5 ± 0.3	0.865 ± 0.004
Dropout 0.1	61.9 ± 1.5	88.2 ± 1.9	13.6 ± 0.3	0.864 ± 0.005
Dropout 0.3	**60.9** **±1.2**	**86.8** **±1.6**	**13.3** **±0.3**	**0.869** **±0.004**
Dropout 0.5	61.6 ± 1.4	87.7 ± 1.8	13.5 ± 0.3	0.866 ± 0.004
Gaussian noise test perturbation	62.3 ± 1.4	88.8 ± 1.8	13.7 ± 0.3	0.862 ± 0.005
ECUSTFD				
Resolution 192 × 192	48.6 ± 1.2	68.8 ± 1.6	12.5 ± 0.3	0.875 ± 0.004
Resolution 224 × 224	**47.2** **±1.0**	**66.8** **±1.4**	**12.1** **±0.2**	**0.883** **±0.004**
Resolution 256 × 256	47.6 ± 1.1	67.3 ± 1.5	12.2 ± 0.2	0.881 ± 0.004
Latent dimension 128	49.0 ± 1.2	69.4 ± 1.6	12.7 ± 0.3	0.872 ± 0.004
Latent dimension 256	**47.2** **±1.0**	**66.8** **±1.4**	**12.1** **±0.2**	**0.883** **±0.004**
Latent dimension 512	47.9 ± 1.1	67.7 ± 1.5	12.3 ± 0.2	0.879 ± 0.004
Dropout 0.1	48.1 ± 1.2	68.1 ± 1.6	12.4 ± 0.3	0.878 ± 0.004
Dropout 0.3	**47.2** **±1.0**	**66.8** **±1.4**	**12.1** **±0.2**	**0.883** **±0.004**
Dropout 0.5	47.8 ± 1.1	67.5 ± 1.5	12.3 ± 0.2	0.880 ± 0.004
Gaussian noise test perturbation	48.4 ± 1.1	68.5 ± 1.5	12.5 ± 0.3	0.876 ± 0.004

Lower values indicate better performance for MAE, RMSE, and MAPE, while higher values indicate better performance for *R*^2^. All values are reported as mean ± standard deviation over 10 runs. The bolded values represent the optimal values.

## 5 Conclusions and future work

The study aimed to address the complex relationship between microbiome composition, athletic performance, and personalized nutrition by introducing the Integrative Microbiome Athletic Performance Optimization Network (IMAPON). This framework integrates microbiome data, athletic performance metrics, and demographic and physiological information to generate personalized dietary recommendations. The IMAPON system is built upon three core modules: the Microbiome Feature Extraction Module (MFEM), the Athletic Performance Prediction Module (APPM), and the Personalized Dietary Recommendation Module (PDRM). The Adaptive Feature Integration Strategy (AFIS) and the Performance Driven Optimization Strategy (PDOS) were developed to improve the framework's adaptability and precision. Experimental results demonstrated that IMAPON effectively identifies latent relationships between microbiome composition and athletic performance, providing actionable dietary interventions that significantly improve performance metrics. The findings highlight the potential of AI driven methodologies to revolutionize sports nutrition by enabling precise, data driven solutions tailored to individual athletes.

Despite the promising results, this study has several limitations that should be acknowledged. The current work is primarily based on computational modeling and benchmark datasets, and does not include human or animal experiments. As a result, the proposed framework has not been directly validated in real world sports nutrition or athletic performance scenarios. The experimental evaluation focuses on the dietary intake estimation component using food image datasets. While this provides a practical and measurable validation of the representation and regression design, it does not fully cover the complete microbiome driven pipeline described in the framework. The absence of publicly available multimodal datasets that jointly include microbiome, dietary, and performance data limits the ability to perform end to end validation of the proposed system. Future work will focus on addressing these limitations by incorporating real world datasets, including longitudinal microbiome and physiological measurements, and conducting validation studies in practical sports nutrition settings. Integrating domain knowledge and knowledge guided modeling techniques may further improve interpretability and biological plausibility.

## Data Availability

The original contributions presented in the study are included in the article/supplementary material, further inquiries can be directed to the corresponding author.
